# Microbial Community Structure of Three Traditional Zambian Fermented Products: Mabisi, Chibwantu and Munkoyo

**DOI:** 10.1371/journal.pone.0063948

**Published:** 2013-05-14

**Authors:** Sijmen E. Schoustra, Chitundu Kasase, Cristian Toarta, Rees Kassen, Alexandre J. Poulain

**Affiliations:** 1 Laboratory of Genetics, Wageningen University, Wageningen, Netherlands; 2 Department of Biology, University of Ottawa, Ottawa, Canada; 3 Departments of Biological Sciences and Food Science and Technology, University of Zambia, Lusaka, Zambia; Wageningen University, Netherlands

## Abstract

Around the world, raw materials are converted into fermented food products through microbial and enzymatic activity. Products are typically produced using a process known as batch culture, where small volumes of an old culture are used to initiate a fresh culture. Repeated over many years, and provided samples are not shared among producers, batch culture techniques allow for the natural evolution of independent microbial ecosystems. While these products form an important part of the diets of many people because of their nutritional, organoleptic and food safety properties, for many traditional African fermented products the microbial communities responsible for fermentation are largely unknown. Here we describe the microbial composition of three traditional fermented non-alcoholic beverages that are widely consumed across Zambia: the milk based product Mabisi and the cereal based products Munkoyo and Chibwantu. Using culture and non-culture based techniques, we found that six to eight lactic acid bacteria predominate in all products. We then used this data to investigate in more detail the factors affecting community structure. We found that products made from similar raw materials do not harbor microbial communities that are more similar to each other than those made from different raw materials. We also found that samples from the same product taken at the same location were as different from each other in terms of microbial community structure and composition, as those from geographically very distant locations. These results suggest that microbial community structure in these products is neither a simple consequence of the raw materials used, nor the particular suite of microbes available in the environment but that anthropogenic variables (e.g., competition among sellers or organoleptic preferences by different tribes) are important in shaping the microbial community structures.

## Introduction

Fermented foods are a significant part of the daily diet of many people in Africa and around the world [1]. During production of a fermented product, microorganisms transform a raw material into a product with increased value, generally by extending the shelf life of the raw materials, by increasing the nutritional value of the product and/or by improving the product’s organoleptic attributes [2], [3]. In most cases, starter culture is added by transferring some of an old batch to fresh raw materials, a process referred to as back-slopping by producers. Back-slopping resembles very closely the batch culture transfer procedure used by microbiologists to study the evolution of microbial populations and communities in the laboratory. When repeated over numerous cycles [1] this process can result in a co-adapted, evolutionarily stable microbial community [4].

The diversity of fermented products found in most African countries is influenced by the available local raw materials and ingredients as well as, to a large extent, by indigenous knowledge and practices. For several African fermented products, the microbial flora has been characterized, critical steps in the process have been identified, and production industrialized. In many cases, the microbes responsible for fermentation are lactic acid bacteria and yeasts that are added to raw materials as a starter culture. These microbes drive the fermentation and hence determine the type of final product and its properties. However, for most African products details on the fermentation process are largely unknown [3], [5], [6], [7], [8].

In Zambia numerous endogenous fermented products are produced, of which Munkoyo, Chibwantu and Mabisi are three important examples of non-alcoholic beverages [5], [6]. These products are consumed on a daily basis by many people and have a great socio-economic and cultural importance in Zambia and Southern Africa. The main motivation for local people to produce these products is their appealing taste, improved nutritional value and prolonged shelf life. In addition, local people claim that the products offer health benefits, such as the prevention and cure of diarrhea. The products are produced at home and almost exclusively by women who sell their product at local markets, providing them with an additional source of income.

The microbial ecology of these three traditional fermented beverages is unknown and forms the focus of this study. We analysed the community structure for products of the same denomination and the same starting raw material (milk for Mabisi, cereal for Chibwantu and Munkoyo) found at different geographical locations and prepared by different tribes. To analyse communities, we used both culture based and non-culture based techniques and this study allows a comparison of the efficiency of these two complementary approaches for the microbial analysis of these products. Our study had two main objectives: (1) to characterise the microbial community structures of the products in an effort to support and develop traditional knowledge, with an eye to eventually facilitating potential process optimisation and economic development, and (2) to obtain some basic understanding of two possible factors governing microbial community structure in these fermented products. More specifically, we asked if starting materials or sampling location is important in structuring the microbial communities within these products.

## Methods

### Preparation and Description of the Products

**Figure 1:**
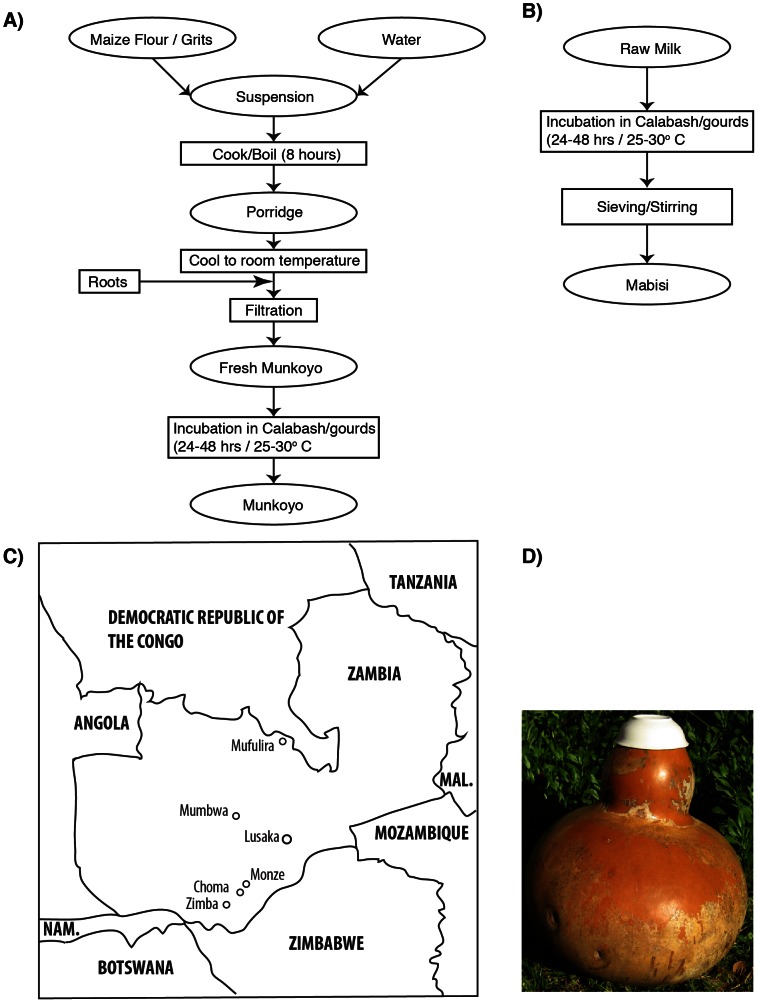
Preparation of the fermented products and sampling locations. A). Process flow for the preparation of Monkoyo. Chibwantu is produced in the same way. If the product is allowed to be fermented for an additional 5 days an alcoholic drink develops that can be kept for prolonged periods of time. B). Process flow of Mabisi. C). Map of Zambia indicating sampling locations (see Table 1). D). Picture of the fermentation vessel (Calabash).

Munkoyo and Chibwantu are non-alcoholic beverages based on cereal raw materials and are produced in a similar way, although the end products have slightly different tastes and organoleptic properties (Figure 1A). Munkoyo is made in the Copperbelt province and northern parts of Zambia; Chibwantu is produced in the Central and Southern areas including those around the capital Lusaka. Both products are made from maize (with sometimes the addition of millet and/or sorghum). Roots of certain plants (Eminia, Rhynchosia and Vigna species; [6]) are added after soaking of the powdered grains and are thought to provide amylolytic enzymes, which make available starch in the cereals by degrading it into maltose (80%), maltotriose (17%) and glucose (3%). The roots are from shrubs that grow as under storey plants in the Miombo Woodland. The roots are tuber-like and fibrous and collectors dig them up and beat them up into fibrous strands that are dried and then used in processing. The fermentation takes place in calabashes (very large fruits that have been hollowed out and can contain up to 40 liters of culture; Figure 1D), which are continuously reused (back slopping). There is a biofilm on the calabash that acts as a starter culture. After around 2 days of incubation at ambient temperatures (ranging from 21°C in winter to 29°C in summer), the fermentation yields the final product, a slightly firm, mildly sour non-alcoholic beverage that is consumed and sold at local markets as an energising and refreshing lunch. The products are also major beverages consumed during special ceremonies. Over the last few years, variants of Munkoyo and Chibwantu (Maheu) have become commercially available via an alternative production process that involves only the addition of enzymes and artificial flavourings. However, no fermentation by live microbial cultures is involved and many people still prefer the traditional Munkoyo and Chibwantu because of their appealing organoleptic properties.

Mabisi is a milk-based product whose fermentation consists of fewer steps than the cereal based products (Figure 1B). Raw milk is transferred into a calabash and fermentation takes place over the course of 2 days at ambient temperatures. The calabash is not cleaned so that it can provide starter culture for the next batch of fermentation. The resulting product has a slightly firm consistency and a mildly sour taste. Mabisi is produced in the southern and central parts of Zambia.

### Sampling

Samples of approximately 250 mL were purchased from producers at local markets (Table 1; Figure 1C). pH of each sample was measured at the sampling site. Samples (2×50 mL and 2×15 mL of the original 250 mL) were then transferred to the laboratory and stored at −20°C and −80°C. Prior to analysis, samples were well mixed by vortexing for at least 45 seconds. Thirty-seven samples were collected in total.

**Table 1:** A total of 37 samples were collected of three product-types; Chibwantu, Mabisi and Munkoyo in various towns across Zambia (Figure 1C).

	SampleID	Product type	pH	Town
1	Chibw-1	Chibwantu	4.2	Mumbwa
2	Chibw-1B	Chibwantu	4.2	Mumbwa
3	Chibw-2	Chibwantu	4.2	Mumbwa
4	Chibw-3	Chibwantu	4.5	Mumbwa
5	Chibw-4	Chibwantu	4.3	Mumbwa
6	Chibw-5	Chibwantu	4.4	Mumbwa
7	Chibw-6	Chibwantu	4.0	Monze
8	Chibw-7	Chibwantu	3.8	Choma
9	Chibw-8	Chibwantu	3.8	Choma
10	Unza-6	Chibwantu	3.8	Lusaka
11	Chibw-9	Chibwantu	4.0	Lusaka
1	Mabisi-1	Mabisi	4.5	Mumbwa
2	Mabisi-2	Mabisi	4.4	Mumbwa
3	Mabisi-3	Mabisi	4.5	Mumbwa
4	Mabisi-1B	Mabisi	4.4	Mumbwa
5	Mabisi-2B	Mabisi	4.5	Mumbwa
6	Mabisi-3B	Mabisi	4.3	Mumbwa
7	Mabisi-5	Mabisi	4.2	Monze
8	Mabisi-6	Mabisi	4.0	Zimba
9	Mabisi-6B	Mabisi	4.0	Zimba
10	Mabisi-7	Mabisi	4.0	Choma
11	Mabisi-8	Mabisi	4.0	Choma
12	Mabisi-9	Mabisi	4.0	Choma
13	Mabisi-10	Mabisi	4.0	Choma
1	Munkoyo-1	Munkoyo	3.5	Mufulira
2	Munkoyo-2	Munkoyo	3.5	Mufulira
3	Munkoyo-4	Munkoyo	3.5	Mufulira
4	Munkoyo-5	Munkoyo	3.5	Mufulira
5	Munkoyo-6	Munkoyo	3.5	Mufulira
6	Munkoyo-7	Munkoyo	3.5	Mufulira
7	Munkoyo-8	Munkoyo	3.5	Mufulira
8	Munkoyo-9	Munkoyo	3.6	Choma
9	Unza-1	Munkoyo	4	Lusaka
10	Unza-2	Munkoyo	3.8	Lusaka
11	Unza-3	Munkoyo	3.8	Lusaka
12	Unza-4	Munkoyo	3.8	Lusaka
13	Unza-5	Munkoyo	4.0	Lusaka

Immediately after sampling, the pH of the sample was measured.

### Culture Based Techniques

Samples were serially diluted in Minimal Salts Medium (Na_2_HPO_4_ 6.7 g, KH_2_PO_4_ 3 g, NaCl 0.5 g, NH_4_Cl 1.0 g, 1000 ml dH_2_O). Dilutions were plated on the following solid growth media: Plate Count Agar (PCA; 0.5% peptone, 0.25% yeast extract, 0.1% glucose, 1.5% agar, pH 7) incubated at 28°C both aerobically and non-aerobically for total aerobic and (facultative) anaerobic counts; PCA plates were incubated aerobically at 42°C for thermophiles; M17 plates (Oxoid) were incubated at 28°C aerobically for lactic acid bacteria; MRS plates (Oxoid) were incubated at 28°C anaerobically for (facultative) anaerobic lactic acid bacteria; YM plates were (0.3% yeast extract, 0.5% peptone, 1% agar, 0.3% malt extract, 1% glucose) at 28°C aerobically for yeast and Violet Bile agar (VB; Oxoid) grown at 28°C for Enterococci. After 4 days of incubation, plates were examined and colonies were counted. From each medium, at least eight colonies were picked capturing all different colony morphologies (if any) and streaked on fresh solid medium for purification. As a result, we collected 30 to 42 bacterial isolates from each sample. These isolates were identified by sequencing part of the V1 to V4 region of the 16S ribosomal DNA gene (see below under PCR amplification).

### DNA Extraction

We designed and optimised DNA extraction procedures for each product. For the cereal based products (Chibwantu and Munkoyo), the protocol for DNA extraction was adapted from Abriouel et al. 2006 and Ampe et al. 1999 [9], [10]. First, we eliminated large particles from 1 gram of product. Samples were spun down at high speed, supernatant was removed and we kept the pellet. We added 500 µl TESL (25 mM Tris, 10 mM EDTA, 20% sucrose, 20 mg/ml lysozyme) and 10 µl metanolysin solution (in water at 1 U/µl), followed by incubation at 37°C for 60 minutes with slight shaking. We added 500 µl GES reagent (5 M guanidium thiocyanate, 100 mM EDTA, 0.5% sarkosyl), cooled on ice for 5 minutes and added 250 µl of cold ammonium acetate solution (7.5 M), followed by gentle mixing. The mixture was held on ice for 10 minutes, spun down and the supernatant removed. We purified the samples by mixing with chloroform-2-pentanol mix (chloroform and 2- pentanol 24∶1) by adding 1∶1 to the supernatant; we spun down and kept the supernatant. We performed a phenol-chloroform purification by adding equal volume of phenol (i.e., tris-saturated phenol-chloroform-isoamylethanol at a ratio of 24∶25:1), vortexed for a few seconds, spun for 2 minutes at 12000 rpm 4°C and transferred the supernatant to a fresh tube. We then added equal volume of chloroform to the supernatant, vortexed for a few seconds, spun 2 minutes at 12000 rpm and 4°C and transferred the supernatant to a fresh tube. We then added 2.5 volumes 100% ethanol, vortexed and precipitated DNA at −20°C overnight. Subsequently, samples were spun for 20 minutes at 12000 rpm and 4°C; the supernatant was removed by aspiration. We washed the DNA by adding 1 mL cold 70% ethanol, spun for 10 minutes at 12000 rpm and 4°C; the supernatant was removed by aspiration and the DNA pelleted was air-dried for 10 minutes at room temperature. Finally, the DNA was dissolved in 10 mM Tris treated with RNAse (10 mM Tris, bring to pH 8.0 with HCl; 1 mM EDTA; RNAse 20 µl/ml).

For the milk-based product (Mabisi) the DNA extraction protocol was performed as follows (adapted from Ercolini et al. 2001, [11]): we started with 1 ml of product in a 1.5 ml microcentrifuge tube and centrifuged the sample at 13,000 × g for 2 minutes to pellet the cells and removed the supernatant. We resuspended the cells in a solution containing 64 µl of a 0.5 M EDTA solution, 160 µl of Nuclei Lysis Solution (Promega), 5 µl RNase (10 mg/ml), 120 µl lysozyme (10 mg/ml) and 40 µl protenase E (20 mg/ml) and incubated for 60 minutes at 37°C. We added 400 µl ammonium acetate (5 M) and cooled on ice for 15 minutes before being spun down at 13,000 × g for 10 minutes. We transferred the supernatant containing the DNA to a fresh 1.5 ml microcentrifuge tube and performed a phenol-chloroform DNA purification as described above.

### PCR Amplification

We used primers 27F (forward primer 5′AGAGTTTGATCMTG GCTCAG′3) and 907R (reverse primer 5′CCGTCAATTCATTTGAG′3) to amplify an 880 bp fragment of the V1 to V4 hypervariable regions on the 16S rRNA gene. We performed this PCR step either for selected purified single isolates, or on the DNA extracted from an entire sample to generate clone libraries. GoTaq and Go buffer supplied by Promega were used for the reaction following the manufacturer’s instructions. For each 25 µl reaction, 2 µl of a 25 mM MgCl_2_ solution was added to the reaction buffer. PCR conditions were as follows: denaturation at 94°C for 2 minutes; then 33 cycles of denaturing at 94°C for 30 sec, annealing at 55°C for 30 sec, elongation at 72°C for 90 sec; followed by a final elongation step at 72°C for 10 minutes.

### Cloning and Sequencing of 16S rRNA Gene PCR Products

PCR products used for cloning were separated by gel electrophoresis (2% agarose). The 880 bp 16S rRNA gene amplicons were extracted and purified from the gel and cloned into vector pSC-A-amp/kan, using a Strataclone PCR cloning kit (Strataclone, Agilent Technologies) according to the manufacturer’s instructions. Eight libraries were created (three for Chibwantu, two for Munkoyo and three for Mabisi). Plasmid DNA was isolated from 96 clones from each library using a PureLink Promega Wizard Magnesil plasmid purification system and underwent ARDRA (see below). Selected clones containing the 880 bp insert were sequenced using the M13f primer (see below). All sequences have been submitted to GenBank, with accession numbers JQ887985 to JQ888112.

### ARDRA

For amplified ribosomal DNA restriction analysis (ARDRA), 48 randomly picked colonies per 16S rRNA gene library were each grown overnight at 37°C in LB containing ampicillin. Plasmid DNA extraction was performed using a Promega Wizard Magnesil plasmid purification system and the insert amplified using primers M13f and M13r. Double restriction digests were made of the PCR products using a combination of EcoRI and AluI for the Chibwantu and Munkoyo libraries and EcoRI and HindIII for the Mabisi libraries. In a total volume of 25 µl, 1 µl of PCR product was digested overnight at room temperature with at least 500 units of each enzyme using appropriate reaction buffer as per the manufacturer’s instructions (New England Biolabs). Restriction patterns were separated on 1.75% agarose gels and stained with ethidium bromide. Each different restriction pattern was defined as an operational taxonomic unit (OTU) (Figure S2). The distribution of OTUs in each library was determined. Two to three representatives of each OTU were sequenced using primers M13f and compared to those in the RDP database [12] or GenBank database using the BLAST algorithm. Diversity indices (Chao1 index, Shannon index, coverage, rarefaction) were computed using MOTHUR [13].

### Phylogenies and Sequences Analyses

Sequencing was performed by Genome Quebec at the sequencing facility of McGill University in Montreal (Canada). All 16S rRNA gene sequences generated were initially scanned for vectors using the PIPELINE function in the Ribosomal Database Project (RDP) [12] and manually inspected. Primers from 16S rRNA gene sequences were removed using Geneious software package primer annotation tool. Chimeric sequences were identified using Mallard, confirmed using Pintail and were discarded [14]. The 16S rRNA sequences were aligned with the latest 16S rRNA gene core alignment downloaded from the SILVA database using MOTHUR [13]. The model of evolution most appropriate to the dataset was determined using jModelTest v.0.1.1 [15]; phylogenies were constructed using FastTree [16] using the appropriate model of evolution and graphically represented using FigTree.

Microbial community structures for each product were compared based on phylogenetic information using the UniFrac metric [17]. For those samples that showed significant difference in the community composition, we performed Jackknife cluster analyses.

Using UniFrac, we computed a (phylogenetic) similarity matrix based on clone library [17], using one clone library (we arbitrarily chose chibwantu1) as a scaling reference. We analysed how phylogenetic similarity correlates with geographical distance through linear regression (Figure S3).

## Results

We collected a total of 37 samples of fermented products from Zambia (Table 1). We characterised the microbial community structures of 3 Zambian fermented products, Mabisi (milk based), Munkoyo and Chibwantu (cereal based) using culture and non-culture based techniques. The pH of the products ranged from 4.0 to 4.5 for Mabisi and Chibwantu and from 3.5 to 4.0 for Munkoyo.

### Characterisation of Isolates and Microbial Communities

**Figure 2:**
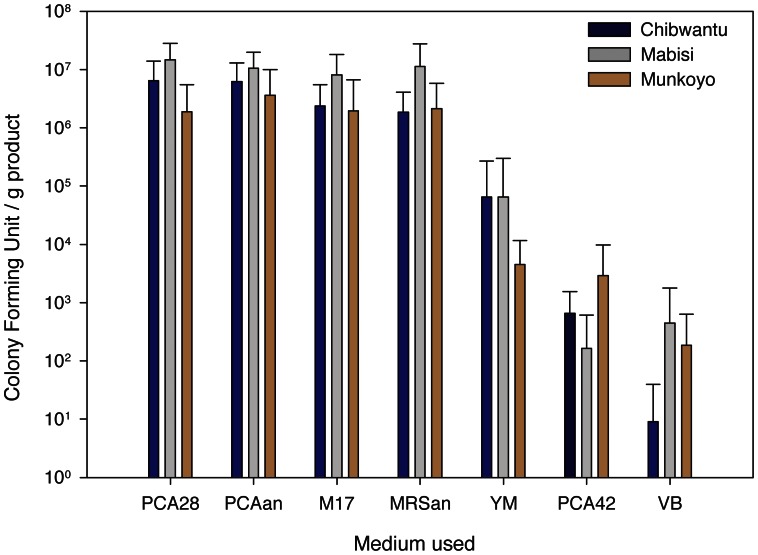
Culture based approach. Viable counts of bacteria present in product samples. Points show the average of all samples of the same product type (Chibwantu, Mabisi and Munkoyo; Table 1). Error bars show standard errors of the mean. PCA28: plate count agar incubated at 28°C for total viable count; PCA42, plate count agar incubated at 42°C, thermophiles; PCAan, plate count agar incubated at 28°C under anaerobic conditions, total (facultative anaerobes), M17: M17 agar, total (aerobic) lactic acid bacteria, MRSan: MRS agar incubated anaerobically, (facultative) anaerobic lactic acid bacteria; YM: total yeast; VB: total Enterobacteria.

For the culture-based analysis we used 5 different growth media (see Methods) which revealed that total viable counts are on the order of 10^7^ cfu/g of product (Figure 2), with highest densities found in Mabisi and lowest in Munkoyo (ANOVA: F_2,35_ = 6.35, p = 0.005). As can be seen from the counts on M17 and MRS agar, facultative anaerobic lactic acid bacteria dominate the culturable components of the communities. This result is consistent with the low pH of the products (Table 1) and suggests that the fermentation vessel is oxygen limited (Figure 1D), providing an environment conducive to acid formation. We occasionally found yeast strains in the products, however, they were not retrieved from every product sample. Counts on VB agar revealed that in all products Enterobacteria were found in low numbers (up to 10^3^ per g).

For each product type, one sample was randomly chosen for culture-based isolation of strains (chibwantu8, mabisi10 and munkoyo5) using purification of single colonies from different selective media (Table 2). From the Petri dishes with the different selective media used to estimate viable counts, a total of 30 to 42 single colonies per product sample capturing all different morphological distinct types were purified and identified by matching 880 bp of their 16S ribosomal DNA sequence to the RDP and NCBI databases (see Methods). Details of the analyses are presented in Table S1. Because we selected strains based solely on their morphology on selective media, and due to bacterial pleomorphism, some strains were retrieved multiple times. *Lactobacillus* was the most abundant genus found, followed by members of the *Leuconostoc*, *Lysinibacillus* and *Bacillus* genera. Only two genera harbouring gram-negative cell wall were found, i.e., *Gluconobacter* and *Enterococcus*. Table S1 provides full details of all sequences obtained.

**Table 2:** List of strains isolated from product samples using culture based techniques.

Product	Isolate	BLAST identity	Abundance (per g of product)
Chibwantu	Chibwantu03	*Bacillus safensis*	10^6^
	Chibwantu07	*Gluconobacter oxydans*	10^6^
	Chibwantu08	*Leuconostoc garlicum*	10^6^
	Chibwantu30	*Bacillus subtilis*	10^6^
	Chibwantu11	*Paenibacillus motobuensis*	10^4^
Mabisi	Mabisi28	*Lactobacillus brevis*	10^7^
	Mabisi19	*Lactobacillus plantarum*	10^7^
	Mabisi09	*Leuconostoc garlicum*	10^6^
	Mabisi11	*Enterococcus durans*	10^6^
	Mabisi15	*Lysinibacillus sphaericus*	10^3^
Munkoyo	Munkoyo09	*Lactobacillus fermentum*	10^6^
	Munkoyo13	*Lactobacillus plantarum*	10^6^
	Munkoyo15	*Bacillus lecheniformis*	10^4^
	Munkoyo31	*Lactobacillus brevis*	10^6^

Based on the dilution at which the strain was isolated, the abundance was estimated. See the Table S1 for full details on the sequences that we obtained to identify each strain.

**Figure 3:**
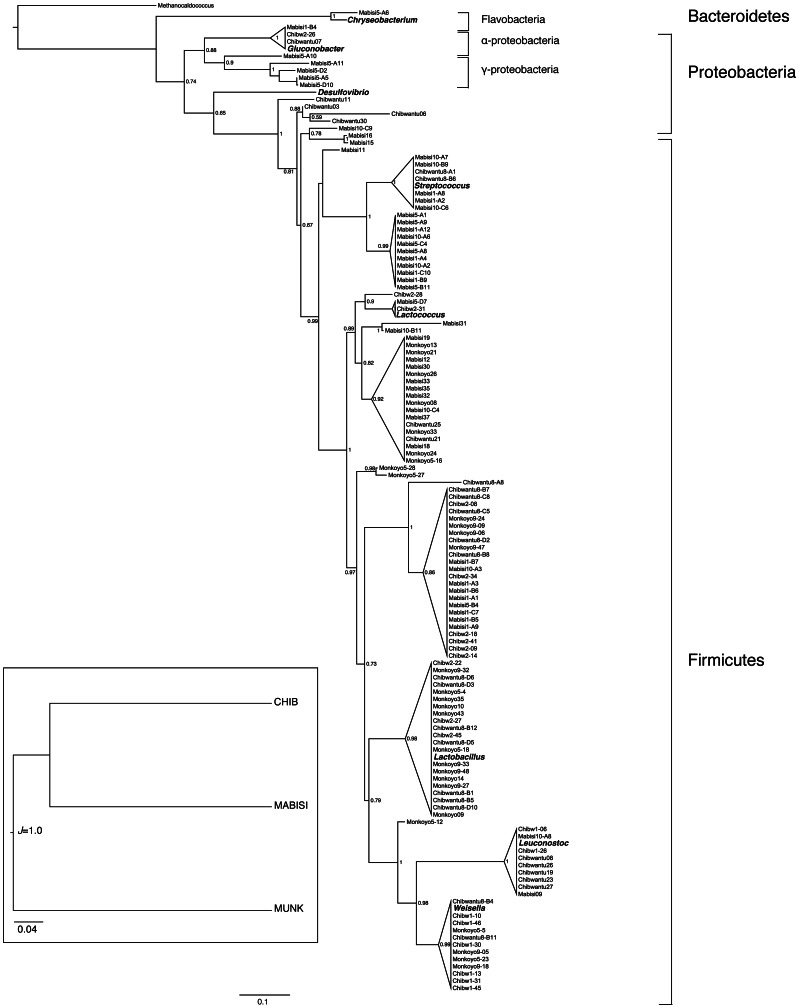
Phylogenetic tree. Maximum likelihood tree of bacterial 16S rRNA gene sequences. Triangle heights are proportional to the number of sequences. Numbers adjacent to each node represent aLRT statistics (SH-like supports). Scale bar shows branch lengths (expected number of substitutions per site). See Tables 2 and 3 and the Table S1 for full strain details. Several known strains (from the NCBI database are shown in bold). The inset shows a cluster analysis based on the product type using all strains in the phylogeny.

We constructed clone libraries for 8 product samples (3 for Mabisi, 3 for Chibwantu and 2 for Munkoyo). Table 3 shows the number of distinct RFLP patterns and the abundance within each pattern. We selected up to 3 clones per RFLP type (11 to 15 clones per library) for sequencing. The identity of clones per observed RFLP pattern is in Table 3 and their phylogenetic relationship is presented in Figure 3 together with several reference strains from the NCBI database. Members of the *Lactobacillus* and *Weisella* genera dominated Chibwantu products; Mabisi products were dominated by members of the *Lactococcus*, *Lactobacillus* and *Streptococcus* genera and Munkoyo products were dominated by members of the *Weisella* and *Lactobacillus* genera. A few gram-negative genera were found, namely, *Acinetobacter*, *Chryseobacterium*, *Acetobacter* and *Gluconobacter* (Table S1, Table 3 and Figure 3). In almost all cases, only one to four key players had abundances greater than 15%. Table S1 provides full details of all sequences we obtained.

**Table 3:** Number of RFLP patterns observed per clone library.

Library	RFLP type	n	Closest relative in the database	fraction (%)
chibw1	1	44	*Weisella confuse*	91.7%
	2	2	*Leuconostoc pseudomesenteroides*	4.2%
	3	1	*Weisella cibaria*	2.1%
	4	1	*Weisella cibaria*	2.1%
	N	48		
chibw2	1	30	*Lactobacillus fermentum*	62.5%
	2	11	*Lactobacillus helveticus*	22.9%
	3	3	*Lactobacillus harbinensis/Lactobacillus casei*	6.3%
	4	2	*Lactobacillus delbreuckii*	4.2%
	5	1	*Acetobacter lovaniensis*	2.1%
	6	1	*Lactobacillus fermentum*	2.1%
	N	48		
chibw8	2	13	*Lactobacillus fermentum/Lactobacillus delbruekii*	28.3%
	3	10	*Lactobacillus delbruekii*	21.7%
	1	3	*Streptococcus macedonicus*	6.5%
	4	3	*Lactobacillus fermentum*	6.5%
	5	3	*Streptococcus macedonicus*	6.5%
	7	3	*Lactobacillus delbruekii*	6.5%
	6	2	*Weisella spp*	4.3%
	8	2	*Lactobacillus fermentum*	4.3%
	9	2	*Lactobacillus delbruekii*	4.3%
	10	2	*Lactobacillus fermentum*	4.3%
	11	1	*Unknown*	2.2%
	12	1	*Weisella cibaria*	2.2%
	13	1	*Lactobacillus fermentum*	2.2%
	N	46		
mab1	1	15	*Lactoccocus lactis*	30.6%
	2	13	*Streptococcus thermophiles*	26.5%
	3	10	*Lactobacillus helveticus*	20.4%
	4	8	*Lactobacillus helveticus; L kefiranofaciens*	16.3%
	5	2	*Lactobacillus helveticus*	4.1%
	6	1	*Acetobacter pasteurianus*	2.0%
	N	49		
mab10	1	26	*Lactococcus lactis*	55.3%
	2	7	*Lactobacillus plantarum/Streptococcus equinus*	14.9%
	3	3	*Streptococcus equinus*	6.4%
	4	3	*Streptococcus thermophiles*	6.4%
	5	2	*Lactobacillus helveticus*	4.3%
	6	2	*Lactobacillus brevis*	4.3%
	7	2	*Unknown*	4.3%
	8	1	*Leuconostoc pseudomesenteroides*	2.1%
	9	1	*Exiguobacterium indicum*	2.1%
	N	47		
mab5	1	28	*Lactococcus lactis*	56.0%
	2	10	*Citrobacter freudii/Acinetobacter ursingii*	20.0%
	3	4	*Acinetobacter spp*	8.0%
	4	3	*Chryseobacterium bovis/Lactobacillus helveticus*	6.0%
	5	2	*Unknown*	4.0%
	6	2	*Lactococcus lactis*	4.0%
	7	1	*Acinetobacter spp*	2.0%
	N	50		
monk5	1	35	*Weisella cibaria/Lactobacillus plantarum/Lactobacillus fermentum*	79.5%
	2	4	*Unknown*	9.1%
	3	3	*Lactobacillus fermentum*	6.8%
	4	1	*Lactobacillus rossiae*	2.3%
	5	1	*Lactobacillus fermentum*	2.3%
	N	44		
monk9	1	36	*Lactobacillus delbruekii*	72.0%
	2	13	*Weisella confusa/Lactobacillus fermentum*	26.0%
	3	1	*Weisella confusa*	2.0%
	N	50		

n indicates the number of times a given pattern was observed. Based on the patterns, 11 to 15 clones were selected for sequencing to reveal the identity of the clones from each library. The data in this table were used to calculate diversity indices (Table 4). All observed RFLP patterns were unique to each sample; the numbering of the RFLP types does not suggest any similarity of RFLP patterns between clone libraries.

We compared the community composition obtained through culture based and non-culture based approaches using the UniFrac significance metric for the 3 product samples (chibwantu8, mabisi10 and munkoyo5). Results showed that the microbial communities identified using culture-based or non culture-based approaches were not different from one another for Mabisi (UniFrac significance; p = 0.13); culture-based and non culture-based approaches yielded different community structures for Chibwantu and Munkoyo (UniFrac significance; p<0.01 in both cases).

Finally, our results revealed that we adequately sampled each of the libraries constructed for each of the products (Table 4). In all cases, using 48 clones was sufficient to capture the variation present. This is supported by Good’s coverage estimates varying from 93% to 98%, by the low values obtained by the Chao1 richness estimators and by the analyses of the rarefaction curves (Figure S1). Overall diversity, estimated by Shannon indices, was low ranging from 0.63 to 0.77 for Munkoyo to 0.39 to 2.20 for Chibwantu. Mabisi exhibited intermediate values ranging from 1.36 to 1.53 (Table 4).

**Table 4:** Diversity indices calculated based on RFLP patterns.

Library	N	n	Chao1	Coverage (%)	Shannon
chibwantu1	48	4	4.5 (4.0–12.3)	96	0.39 (0.10–0.67)
chibwantu2	48	6	6.5 (6.0–14.3)	96	1.10 (0.81–1.38)
chibwantu8	46	13	13.6 (13.1–20.1)	93	2.20 (1.93–2.47)
mabisi1	49	6	6*	98	1.53 (1.35–1.70)
mabisi10	47	9	9.25 (9.0–13.8)	96	1.53 (1.20–1.86)
mabisi5	49	7	7*	98	1.36 (1.06–1.66)
munkoyo5	44	5	6 (5.1–18.5)	95	0.77 (0.44–1.09)
munkoyo9	50	3	3*	98	0.63 (0.44–0.83)

Numbers between brackets show 95% confidence intervals. N: number of patterns analysed; n: number of observed unique patterns; Chao: estimation of the total number of unique types in the population; * indicates that the rarefaction curve reached saturation; coverage: fraction that our sampling covers from total number of types; Shannon: Shannon diversity index.

### Microbial Community Structure Comparisons

We used UniFrac tools to compare microbial community structures of the various fermented products investigated using phylogenetic information [16]. When compared using the UniFrac significance metric, community structures of Munkoyo, Mabisi and Chibwantu were significantly different from each other (Table 5A). The results of the cluster analysis (Figure 3 inset) revealed that in all cases, Munkoyo clustered away from Chibwantu and Mabisi, suggesting that the communities in Chibwantu and Mabisi are more similar to each other than the communities in Munkoyo. A Jackknife analysis provides further support for this result: Munkoyo clustered away from Chibwantu and Mabisi 100% of the time. Due to lack of statistical power, Mabisi and Chibwantu differences could only be retrieved 15% of the time with the Jackknife analysis.

**Table 5:** UniFrac significance of comparisons of microbial communities based on clone library sequences in the phylogeny presented in Figure 3.

A	Chibwantu	Mabisi	Munkoyo	
Chibwantu	0	0.03	0.03	
Mabisi	0.03	0	≤0.03	
Munkoyo	0.03	≤0.03	0	
**B**	**Chibw1**	**Chibw2**	**Chibw8**	
Chibw1	0	≤0.03	≤0.03	
Chibw2	≤0.03	0	≤0.03	
Chibw8	≤0.03	≤0.03	0	
**C**	**Mab1**	**Mab10**	**Mab5**	
Mab1	0	0.99	0.06	
Mab10	0.99	0	0.24	
Mab5	0.06	0.24	0	
**D**	**Choma**	**Monze**	**Mufulira**	**Mumbwa**
Choma	0	≤0.06	0.18	1
Monze	≤0.06	0	≤0.06	≤0.06
Mufulira	0.18	≤0.06	0	0.06
Mumbwa	1	≤0.06	0.06	0

Comparison based on (A) all sequences pooled per product; (B) only the three Chibwantu libraries; (C) only the three Mabisi libraries; (D) all sequences pooled per sampling location. Values show Bonferroni corrected p-values for multiple comparisons.

**Figure 4:**
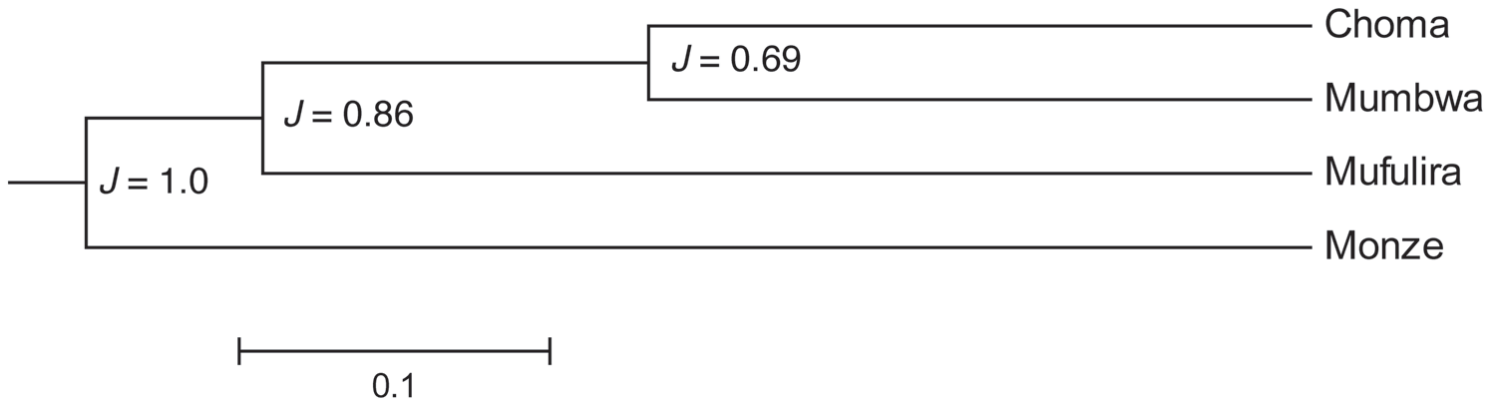
Effect of sampling location on product similarity. Jackknife cluster analysis based on sampling location using pooled data over all clone library sequences. Numbers adjacent to each node represent the estimated support value (%) after 100 permutations.

We further analysed libraries of the same product type but from different markets or vendors and found that most microbial communities were significantly different from one another except for Mabisi (Table 5 B and C). It might be expected that, for a given product, microbial communities collected in close proximity would be more similar to each other than those from more distant locations. This was apparently not the case, at least for Chibwantu and Mabisi. Libraries of samples taken at geographically proximal locations for these products did not group together. A cluster analysis on all product samples, pooled based on their sampling location, also found that samples collected in proximity to each other did not group together. For example, the distance between the towns of Choma and Monze is about 50 km and that between Choma and Mumbwa is 350 km (Figure 1D) but the microbial community of samples collected in Choma and Monze are apparently the most different in our analysis (Figure 4; Table 5D). Furthermore there is no significant negative relationship between the geographical distance at which the samples were collected and the similarity of the microbial community composition, as would be expected if microbial community similarity declines with distance. Using one library (Chibwantu1) as a reference and pooling all other clone libraries, we found a marginally significant positive relationship between phylogenetic similarity and geographical distance, just the opposite to our expectation (Figure S3).

## Discussion

This study had two main objectives. Firstly, we characterised the microbial community structures of the products using culture based and non-culture based techniques, our primary aim being to provide and promote indigenous knowledge for potential commercial applications. The three products under investigation are widely consumed in Zambia and such basic, fundamental knowledge on the natural history of the microbial communities in these products forms a necessary first step towards understanding the details of the fermentation process and how this might be exploited to support cultural and economic development in the future. Secondly, we provide some preliminary investigations of two potential factors that shape the structure of these communities by asking whether starting raw materials or sampling location contribute to the structure of microbial communities within these products.

### 1. Microbial Community Composition

The analysis of the microbial communities in the products through culture based approaches revealed that the community in each of the product is represented by 4 to 13 dominant taxa of mainly lactic acid bacteria of genera such as *Lactobacillus*, *Lactococcus*, *Streptococcus*, *Weisella*, and *Leuconostoc*. This was expected since all products exhibited a pH of around 3.5 to 4.5. *Lactobacillus* and *Streptococcus* species are homofermenters producing lactic acid as major end product. *Weisella* and *Leuconostoc* species are heterofermenters that apart from acids also produce ethanol and CO_2_ [3], [8]. Members of the *Acetobacter* genus are the dominant gram-negative bacteria found in these products and can convert ethanol into acetic acid when enough oxygen is present [3]. The community we found is consistent with that found in other milk based products from Southern Africa [18] and other Southern and Western African cereal based products [3]. Viable counts were in the order of 10^7^ per gram of product, which is in line with viable counts found in other studies (e.g. [18]). The detection limit in our non-culture based analysis is on the order of 1 to 2% of the most abundant type since we analysed 48 RFLP patterns for each clone library.

We compared, for the same product, the microbial community structures obtained using culture and non-culture based approaches. We found that we the microbial communities among samples within the two cereal based products (Munkoyo and Chibwantu) differed significantly from each other, while those for the milk based product (Mabisi) did not. While both methods give similar results at the level of the types of species found in the communities, this suggests that the laboratory culture media (solid plate count agar, MRS agar and M17 agar) quite strongly mimic the conditions in the milk products but that certain key nutrients or other conditions of the cereal products are not met. The non-culture based approach likely gives a more accurate result since this method only relies on a proper DNA extraction of all bacterial DNA from the sample and unbiased PCR [9], [11]. Although it is likely that additional members may have been found for each community if we had used second generation sequencing techniques to our samples [19] the analysis of our diversity indices, rarefaction curves and estimated coverage suggests that this result is likely to be fairly robust. We conclude that there can be substantial variation among producers in the microbial community of cereal-based products despite those products having the same name and rather similar functional and organoleptic properties, although the underlying reasons why this is so remain unclear at this time.

### 2. Comparison of Microbial Community Structures between Samples Based on Differences in Raw Materials and Sampling Location

It seems reasonable to expect that the raw materials and the different fermentation procedures used to create environments with different physical properties should support and promote a different combination of microbial species. However we found only partial support for this hypothesis. Although both UniFrac significance tests and cluster analyses showed that the different products (Mabisi, Chibwantu and Munkoyo) have unique microbial communities, Munkoyo communities clustered away from those of both Mabisi and Chibwantu. This result is surprising because we would have expected Munkoyo and Chibwantu, being both cereal based, to be more similar to each other than either would be to Mabisi, which is milk based. Nevertheless the pH of the more similar communities, Mabisi and Chibwantu, is also very similar lending further evidence that the microbial communities are indeed quite similar. The proximate reasons for this outcome are not directly evident but may result from subtle differences in the preparation of Munkoyo and Chibwantu (Figure 1A). The boiling time for the cereal/water suspension is slightly longer for Munkoyo than for Chibwantu, for example, and the roots added to the fresh Munkoyo and Chibwantu could be slightly different since the products are made in different parts of Zambia. Thus it may be that small differences in preparation can lead to large differences in their component microbial communities of very similar products.

We also found little support for the idea that geographical isolation among producers leads to more divergent microbial communities within a product, as would be expected to be the case if raw materials and climate are more similar for samples collected in close proximity compared to those that are more distant. The UniFrac significance tests and cluster analysis based on where the populations were sampled (Figure 4, Table 5) showed no signature of geographical location on community composition. This was true whether we examined all samples together or between samples of the same product (Figure 4, Table 5, Figure S3). Nevertheless there can be substantial differences in microbial community composition between producers of the same products (Tables 3 and 5), and so the question remains as to how such different communities give rise to what are recognized by consumers as effectively the same product. It may be that other factors such as soil type, microbes already present in the raw materials, or ambient climatic conditions play important roles but further tests of these hypotheses requires additional sampling and data collection.

Another, intriguing, possibility is that microbial communities of products of the same denomination are different because producers who sell their product in close proximity of each other try to achieve slightly different tastes. These different tastes, and the preferences of consumers, could be a result of the tribal background of the producer and their target consumer. This interpretation gains some support from the fact that Mumbwa, a place where we collected multiple samples, is a regional centre where different tribes congregate, although it belies the reported claims of local producers that rather uniform production procedures are employed around the country. After analysing the samples, we revisited the sampling locations to discuss the results with local producers (in Mumbwa and Lusaka). Our enquiries revealed that producers in the same town try to make their product be slightly different from others by slight modifications to the production procedure, mainly in the alteration of the incubation times at the various steps (Figure 1A,B). It may be that competition for customers among producers leads to the production of slightly different products, which would be reflected in a form of character displacement at the level of the microbial community. Given that slight differences in production methods seem to have a large effect on microbial community composition between cereal-based products, it does not seem too far-fetched to think that they might also have noticeable effects on community composition of the same product as well. Further research is required to investigate this.

The hypothesis that anthropogenic factors are important shapers of microbial communities in fermented food products is particularly exciting and could potentially explain some of the other unexpected results we obtained and deserves future exploration. Different tribes prepare the two cereal base products, Munkoyo and Chibwantu, with Munkoyo being produced primarily in the northern part of Zambia by Bemba tribes and Chibwantu being produced in central and southern Zambia by the Kaonde, Ngoni and Tonga tribes. Cultural traditions vary across Zambia [20] and variation in these traditions could become reflected in different microbial community structures of the products they produce. The role of anthropogenic and other site-specific factors in governing microbial community structure can be investigated further by including a sociological approach involving careful interviews of producers to elucidate exactly what the differences in production procedure are and investigating whether these can be linked directly to observed differences in microbial community structure.

### 3. Socio-economic Implications

Gaining insights into the microbiology of traditional fermented African products also has practical implications both at local and broader scales. The three fermented products that are the focus of this study are widely consumed across Zambia, however their microbial composition has to date not been studied. Knowledge on the microbial composition and factors that affect this composition will promote and improve indigenous knowledge and practices, enhance food security for local communities at low costs [21] and provide opportunity for economic development.

Fermented products are known to promote human health because they are nutritious and safe: the microbial communities in the products are highly stable and are able to withstand an invasion by pathogenic bacteria and in some cases detoxify the raw materials [8], [21], [22]. We found that the microbial communities in the products is predominated by lactic acid bacteria, which supports the claim by local knowledge that consumption of Mabisi, Chibwantu and Munkoyo promotes human health. In particular it is claimed that these products suppress diarrhea and are thought to mitigate symptoms of HIV/AIDS and hypertension and anti-allergen properties [23], although more direct tests of these claims is warranted. Nevertheless, lactic acid bacteria are known for their specific antimicrobial properties that determine their bio-preservation properties in foods, including the production of organic acids, hydrogen peroxide, carbon dioxide, diacetyl and broad-spectrum antimicrobials such as anti-competitor toxins [8]. Organic acids such as lactic and acetic acids are potent antimicrobials in low pH environments, [8], [24] and the antimicrobial properties of the products we studied are recognized by local knowledge in Zambia where they are promoted for their medicinal properties. Our own pilot studies with Mabisi indeed suggest that pathogen-like strains such as *Listeria innocua*, *Escherichia coli* and *Staphylococcus epidermidis* are unable to perturb the stable resident community in the products, even when inoculated at densities of 10^6 ^per gram of product (data not shown). The precise mechanisms involved in preventing this invasion into the microbial community – whether it is the lower pH or perhaps the production of bacteriocins or other anticompetitor toxins – remains an avenue for further investigation. Advancement of knowledge on these traditional products, including how exactly their safety is ensured, could further point to ways to optimize existing fermentation processes, to novel processes which may provide new strategies to fight more serious infections of worldwide concern, and facilitate the effective marketing the traditional version of the products, which can generate income for local communities [1], [25].

### 4. Traditional Fermented Products as Model for the Study of Ecosystem Ecology and Evolution

Microbial systems are now routinely used to study the basic evolutionary processes of adaptation and diversification in well-defined communities of one or at most a few species [4], [26], [27], [28], [29], [30]. Ideally, scientists studying evolution would like to predict the ecological properties and evolutionary dynamics of highly complex ecosystems as well but the ability to scale up their inferences from simple communities in the lab to more complex ones in nature remains limited. Needed to fill this gap are experimental model systems for studying long-term whole community evolution, but these are almost entirely lacking [31], [32]. The products used in this study may be such a system. Since the product is produced by repeated propagation of starter culture and without exchange with other producers, the constituent microbial communities have been evolving independently from each other probably for at least hundreds, if not thousands, of microbial generations. In essence, then, each product from a different producer represents an independent instance of adaptive community evolution towards a defined end-point – the fermented product – with known ecological function. These functions include both nutritional content, appeal to the consumer, and safety. This system thus opens new avenues for research on how complex communities with known functional properties are assembled and evolve.

## Supporting Information

Figure S1 **Rarefaction curves of clone libraries based on RFLP patterns.**
(TIF)

Figure S2 **RFLP patterns of 48 clones from the Mabisi1 clone library, digested with EcoRI and HaeIII.** The ladder is a 100 bp ladder. From these patterns, the number and frequency of the different distinct patterns observed here was used to calculate diversity indices. In this library, we observed 6 different patterns. To the right of each row is the non-digested negative control.
(TIF)

Figure S3 **Phylogenetic similarity of microbial communities as a function of geographical distance.** We examined the relationship between the geographical distance at which the samples were collected and the similarity of the microbial community composition; this similarity was computed using the phylogenetic tree using one library (Chibwantu1) as a reference. Pooling all clone libraries, we found a marginally significant positive relationship between phylogenetic similarity and geographical distance (multiple r^2^ = 0.5385, F_1,5_ = 5.83, p = 0.060), Since one library is used as a scaling reference in each comparison, the analysis for each product type could only be done with the Chibwantu and Mabisi libraries, for which we each analysed three samples, leaving us with only two points for each library and thus no statistical power to detect a relationship between phylogenetic similarity and geographical distance for each product type separately. Nevertheless, this analysis confirms the analysis described above which showed that phylogenetic similarity is lowest for microbial communities in samples collected in close proximity. A generalised linear model on the pooled dataset with phylogenetic similarity as the dependent variable and geographic distance and product type as independent variables revealed no significant effect of either independent variable nor of their interaction on the variation in phylogenetic similarity.
(TIF)

Table S1 **Isolates and clones most closely related relatives in NCBI and RDP databases.**
(XLSX)
