# Supplementary figures and images for: Microbial Community Structure of Three Traditional Zambian Fermented Products: Mabisi, Chibwantu and Munkoyo
Source: PLoS One. 2013 May 14;8(5):e63948. doi: 10.1371/journal.pone.0063948 (PMC3653860; doi:10.1371/journal.pone.0063948)

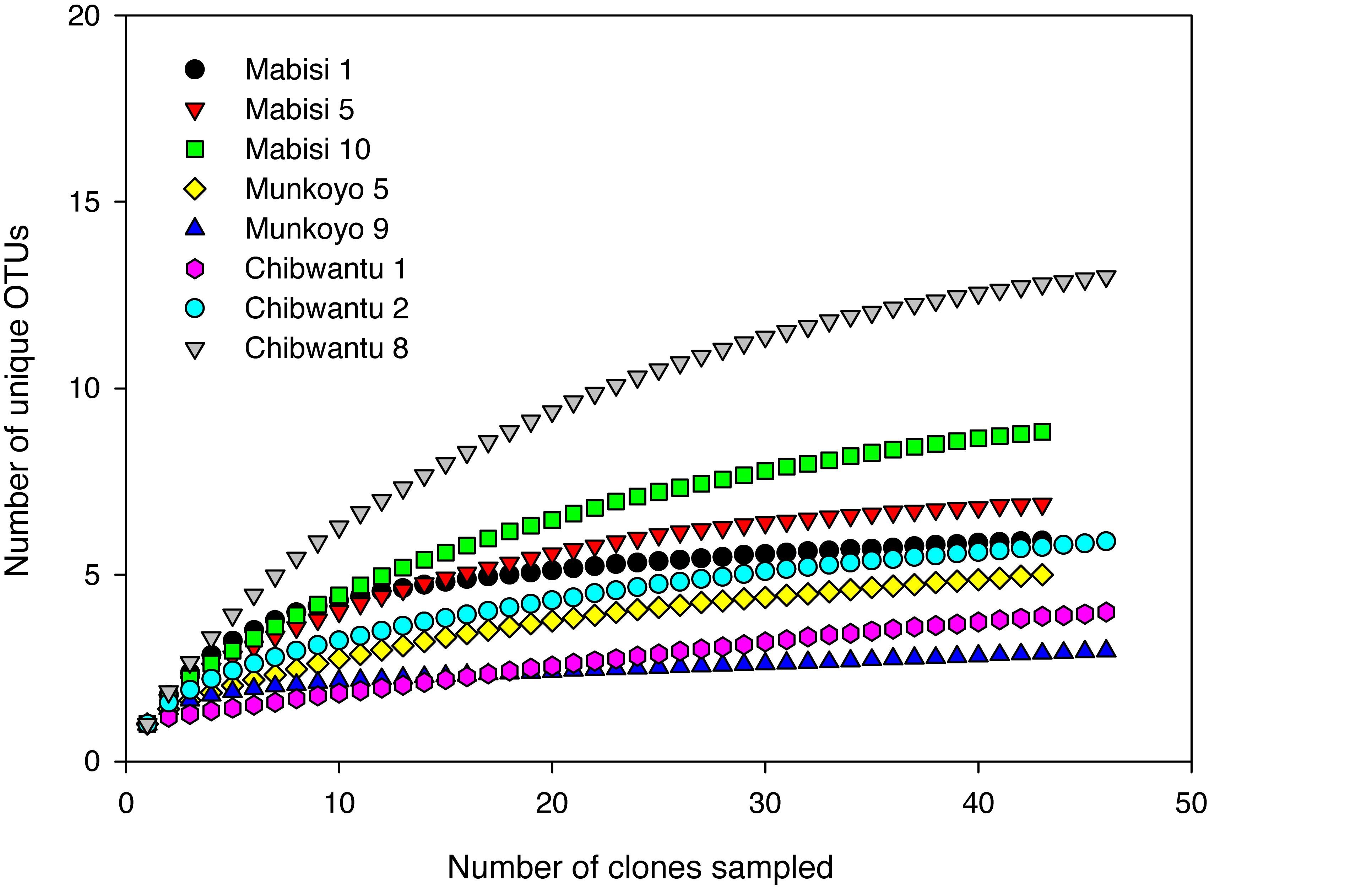

Supplement: Figure S1 — Rarefaction curves of clone libraries based on RFLP patterns. (TIF) [file pone.0063948.s001.tif]

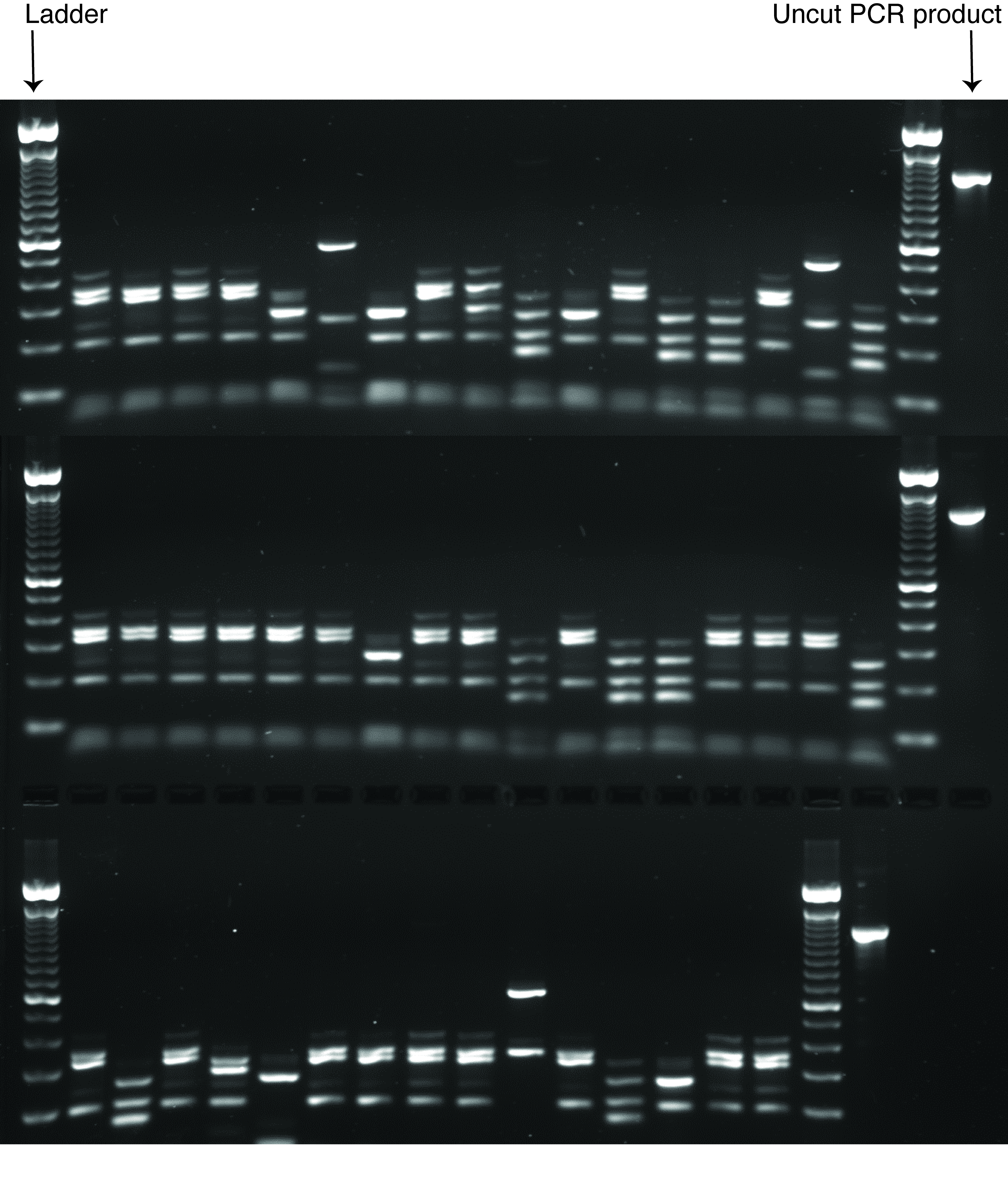

Supplement: Figure S2 — RFLP patterns of 48 clones from the Mabisi1 clone library, digested with EcoRI and HaeIII. The ladder is a 100 bp ladder. From these patterns, the number and frequency of the different distinct patterns observed here was used to calculate diversity indices. In this library, we observed 6 different patterns. To the right of each row is the non-digested negative control. (TIF) [file pone.0063948.s002.tif]

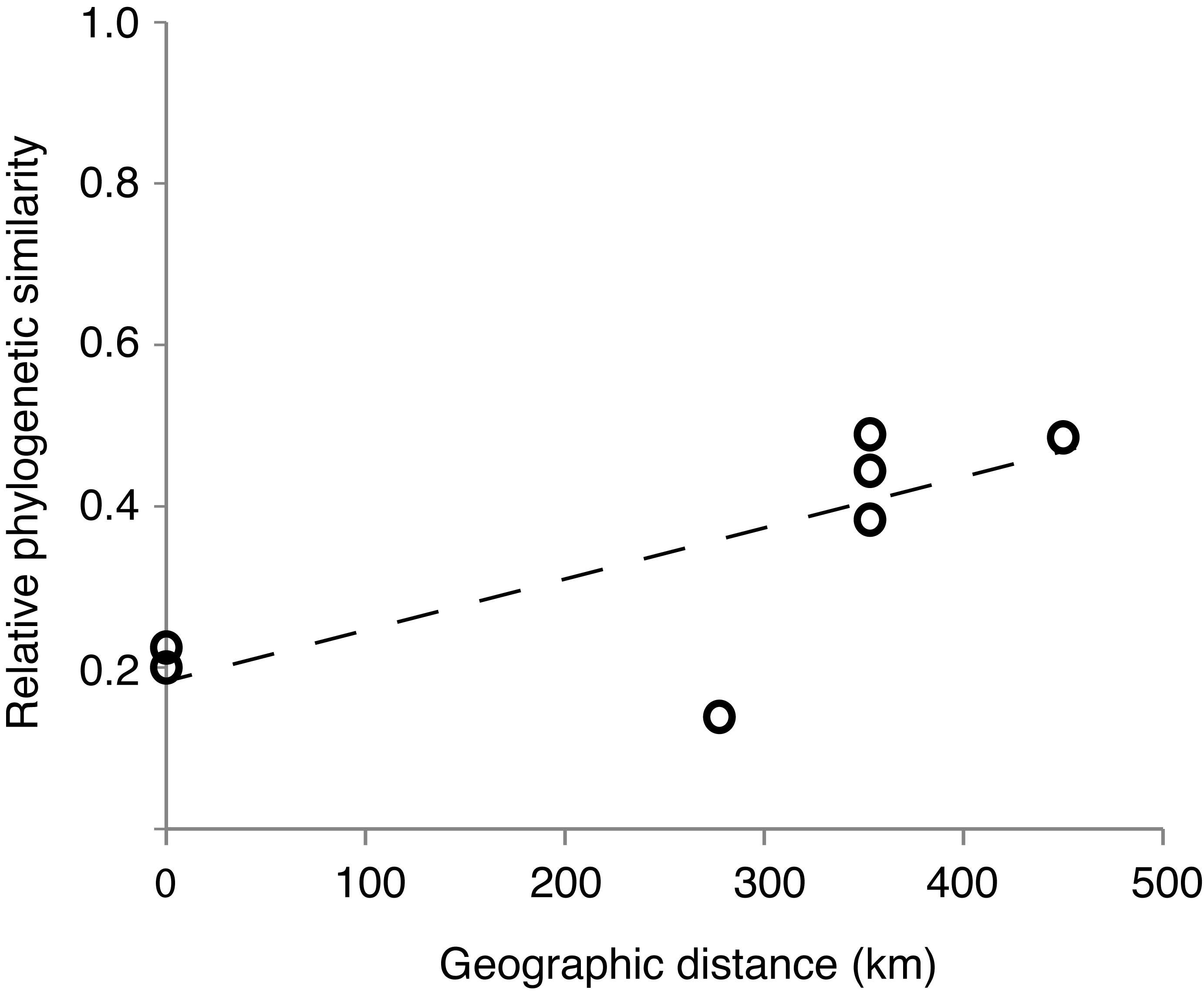

Supplement: Figure S3 — Phylogenetic similarity of microbial communities as a function of geographical distance. We examined the relationship between the geographical distance at which the samples were collected and the similarity of the microbial community composition; this similarity was computed using the phylogenetic tree using one library (Chibwantu1) as a reference. Pooling all clone libraries, we found a marginally significant positive relationship between phylogenetic similarity and geographical distance (multiple r2 = 0.5385, F1,5 = 5.83, p = 0.060), Since one library is used as a scaling reference in each comparison, the analysis for each product type could only be done with the Chibwantu and Mabisi libraries, for which we each analysed three samples, leaving us with only two points for each library and thus no statistical power to detect a relationship between phylogenetic similarity and geographical distance for each product type separately. Nevertheless, this analysis confirms the analysis described above which showed that phylogenetic similarity is lowest for microbial communities in samples collected in close proximity. A generalised linear model on the pooled dataset with phylogenetic similarity as the dependent variable and geographic distance and product type as independent variables revealed no significant effect of either independent variable nor of their interaction on the variation in phylogenetic similarity. (TIF) [file pone.0063948.s003.tif]
